# Dr. Cayley, on Aneurism

**Published:** 1807-01

**Authors:** G. Cayley

**Affiliations:** Ripon


GENTLEMEN,

SHOULD you think the following case deserving publicity, I request you will be so obliging as to give it a place in the next number of your Journal; and, should it throw fresh fresh light upon the mode of treating Aneurism, I shall be gratified in having made the trial, and in having communicated the result to the public.

Mr. Samuel Wilkinson, a very respectable farmer at Hartwith, twelve miles west from this place, called me in to visit him, in consultation with Mr. Strother, surgeon, from Pately, on account of an accident he had received on his right leg thirteen weeks before. He was assisting his people to sheer his sheep, when one of the animals kicked the sheers out of his hand, and forced one of the points into his leg a considerable depth, and a copious hæmorrhage ensued from the wound immediately, which was, however, at length restrained by pressure of the thumb only, until Mr. Strother was brought from Pately, a distance of three miles, and the hæmorrhage did not return. It was the arteria tibialis antica that was wounded; it occasioned considerable inflammation and pain, nor was he able to point his foot to the ground; in about ten days, however, the wound healed by the first intention, and Mr. Strother then discontinued his visits, thinking his patient was doing well, being able to walk about, though not without pain and some apprehension of a recurrence of hæmorrhage.

Betwixt five or six weeks afterwards, Mr. Strother was again called in, as a considerable tumour had formed on the part where the wound had been inflicted, which occasioned a great deal of pain and constitutional irritation, he was not able to use his leg; and was dreadfully tormented with spasms in the whole of that limb. They had poulticed it for a considerable length of time previous to their sending for Mr. Strother a second time, who ordered it to be fomented every three hours with hot fomentations, and afterwards to be poulticed. This plan was strictly pursued for a long time, without producing any change that appeared favourable; the skin became a deep red, and appeared rather thinner than in its healthy state. Mr. Strother now resolved, that if the tumour should not break, he would at his next visit, on the 25th of August, lay it open. On his arrival he proceeded to examine it; but to his great surprise and mortification he found it to be an aneurism, and told his patient his opinion, and the probable result; which gave Mr. Wilkinson considerable uneasiness, as he dreaded the operation, and was fearful that the artery should burst, and carry him off.

A simple poultice was now ordered to be applied, and I was desired to attend on the 27th, without having been informed informed of the nature of the case. On my inspecting the leg, I did not hesitate to pronounce it an aneurism of the tibialis antica. The sac was of a considerable size, the pulsation throughout the whole was not only tangible, but visible across the room; the blood was very readily forced into the artery by gradual pressure, which occasioned a great deal of pain. When the pressure was removed, the blood instantaneously and forcibly returned into the sac, and proved to me at once what was the nature of its contents.

I informed Mr. W. in as cautious a manner as possible, of the probable result, and the possibility of an operation; the uneasiness of his mind is not to be described, and he laboured under a considerable degree of irritative fever.

Having now ascertained the nature of the case, and proved that the blood was returnable into the artery, without difficulty, I proposed the following novel mode of treatment, with a view, should it not succeed, to gain time and to prepare him more fully for the operation, to which he was very averse.

I ordered a piece of strong thick pasteboard, near three inches in length, and one and a half in breadth, to be laid over the tumour, and gradual pressure to be applied until the blood was forced into the artery; a tight bandage was then applied, and the leg was constantly bathed with a strong solution of ammonia muriata in vinegar and water, and the bandage tightened whenever it had become slack. Mr. Strother having very politely acquiesced in this plan, we agreed that the leg should not be examined again in less than ten or twelve days, during the whole of which time, it was kept moistened day and night with the above lotion.

On the 6th of September we returned; (Mr. Strother having visited him occasionally in the interval) during this period, he felt various sensations, sometimes a great deal of pain, sometimes none at all; at one time considerable numbness, with a prickling sensation was felt in the whole limb, from the posterior part of the thigh to the toes, which he could not move, nor his ankle joint. He took all this time cinchona, nitre and opium, with an occasional laxative, and abstained from all stimulating liquors.

We now proceeded to examine the leg, not without some fear as to the result of our new mode of treatment; but it is not easy to describe our feelings, when the injured parts came to view; the tumour was completely removed; inflammation very trifling, and not the slightest pulsation was to be felt, with little or no pain, even upon the hardest pressure; the cuticle had a number of pustules, discharging a small quantity of matter; they were very superficial, and were occasioned no doubt by the lotion, and were healed in a few days with the ungt. ceruss.

The bandage was again applied, and he was ordered the cinchona with the sulphuric acid, and an opiate at night. The ankle to be rubbed with the volatile liniment, and to be moved in different directions.

Sept. 22d. We visited him again, and found his general health very much improved, and his leg had recovered its usual healthy appearance; he had not yet ventured to use it, which I desired him to do by degrees.

On the part where the wound was inflicted, there was a small colourless elevation, without the slightest pain; this I considered as a mere thickening of the integuments; it was perfectly firm to the touch. Some officious person had, however, brought one of those pests of society to visit him, who go by the name of far Doctors, Stubbs is his name, he lives at Bulmer, near Castle Howard; this sapient Doctor assured Mr. Wilkinson, that the swelling contained a large collection of matter, covered with proud flesh and skin; that he had been very ill treated by the medical gentlemen who attended him; that the parts should be eat away by caustics, and that if he would put himself under his (Stubbs') care, he would cure him in a short time. This Mr. W. declined doing, and the self-dubbed Doctor retired in an angry mood, not expecting to meet with so cool a reception.

It is to be hoped that the Medical Reform will put an entire stop to the wide spreading mischiefs of these abominable pretenders to so difficult an art as that of surgery. I assured Mr. W. that he had nothing to apprehend, and he was well pleased that he had not been made the dupe to this impostor.—The bandage was again applied, he was ordered a more generous diet with wine, and to walk about, and that I would visit him again in a fortnight.

On the 6th of October I again saw him with Mr. Strother; all was doing perfectly well, but he was too timorous of using his leg, though he could move the ankle joint perfectly well; I begged he would use his leg more than he did.

Oct. 13th. I received a letter from Mr. Strother, who says, "Mr. Wilkinson's leg continues to mend; he walks about a little every day, but feels his leg weak yet; complains of an uneasy rheumatic affection in the knee. Ordered dered the knee to be rubbed with volatile liniment and laudanum; the bandage to be continued over the wounded part only, with thin pasteboard over it.

It now only remains for me to give my reasons for having recommended the above treatment; if it has ever been adopted, or should ever be adopted under similar circumstances, I shall feel myself infinitely obliged to the Gentlemen to whose care such cases may be submitted, to publish the result in the different Medical Journals.

I was certain that the sac would go on increasing until it would burst, unless some speedy method should be adopted to prevent it;—the operation for aneurism was much dreaded by our patient and his family. The communication betwixt the sac and the artery was free; I could easily force the contents of the former into the latter, and by pressure confine the blood in the artery; this led me to recommend continued pressure, in hopes too of bringing the wounded parts of the artery into contact, and thereby to promote cicatrization; and if by pressure, the diameter of the artery should be diminished, or obliterated, the anastamozing vessels would take up the blood, absorption would be promoted, the parts would be strengthened, and a firm union take place betwixt the wounded artery and the parts contiguous; and I hope the result has justified my conduct.

I am, &c.

G. CAYLEY, M. D.

*Ripon, Oct.* 13, 1806.

---

### *To the Editors of the Medical and Phyfical Journal.*

Gentlemen,

